# Polonium and Lung Cancer

**DOI:** 10.1155/2011/860103

**Published:** 2011-06-23

**Authors:** Vincenzo Zagà, Charilaos Lygidakis, Kamal Chaouachi, Enrico Gattavecchia

**Affiliations:** ^1^Department of Territorial Pneumotisiology, AUSL of Bologna, 40124 Bologna, Italy; ^2^Regional Health Service of Emilia Romagna, AUSL of Bologna, 40124 Bologna, Italy; ^3^Université Paris XI, DIU tabacologie, 75012 Paris, France; ^4^Complex Unit of The Institute of Chemical, Radiochemical, and Metallurgic Sciences University of Bologna (SMETEC), 40126 Bologna, Italy

## Abstract

The alpha-radioactive polonium 210 (Po-210) is one of the most powerful carcinogenic agents of tobacco smoke and is responsible for the histotype shift of lung cancer from squamous cell type to adenocarcinoma. According to several studies, the principal source of Po-210 is the fertilizers used in tobacco plants, which are rich in polyphosphates containing radio (Ra-226) and its decay products, lead 210 (Pb-210) and Po-210. Tobacco leaves accumulate Pb-210 and Po-210 through their trichomes, and Pb-210 decays into Po-210 over time. With the combustion of the cigarette smoke becomes radioactive and Pb-210 and Po-210 reach the bronchopulmonary apparatus, especially in bifurcations of segmental bronchi. In this place, combined with other agents, it will manifest its carcinogenic activity, especially in patients with compromised mucous-ciliary clearance. Various studies have confirmed that the radiological risk from Po-210 in a smoker of 20 cigarettes per day for a year is equivalent to the one deriving from 300 chest X-rays, with an autonomous oncogenic capability of 4 lung cancers per 10000 smokers. Po-210 can also be found in passive smoke, since part of Po-210 spreads in the surrounding environment during tobacco combustion. Tobacco manufacturers have been aware of the alpha-radioactivity presence in tobacco smoke since the sixties.

## 1. Introduction

WHO has declared a tobacco epidemic, indicating the spread of smoking dependency, which affects 1.3 billion people worldwide and results in 5.4 million tobacco-related deaths each year. If this trend continues, there will have been 10 million deaths by 2030 [1]. Smoking-related diseases include lung, esophagus, and pancreas cancer, cardiovascular diseases, COPD, pneumonia, sudden infant death syndrome, prematurity, and deaths caused by fires from cigarette stubs [2]. In Europe 650000 people die each year from smoking-related diseases.

Every year, approximately 11 million people are diagnosed with cancer worldwide; 8 million of them will die [3]. Cancer is a significant public health problem in Europe as well. In 2000, 1122000 deaths from cancer were registered in the 25 countries of the European Union (EU). From 1990–1994 to 2000–2004, mortality for all types of cancer in the EU declined from 185.2 to 168.0 per 100000 (world standard, −9%) in men and from 104.8 to 96.9 (−8%) in women [4, 5].

Tobacco smoking is a risk factor for six out of eight main death causes all over the world; with lung cancer being one of the six causes, tobacco represents the most important one [6, 7]. Each year 1.35 million new cases are diagnosed, which represents more than 12% of all the new cancer cases [8]. Furthermore, smoking is responsible for 1.18 million deaths from cancer (17.6% of the world total) [9], of which 21400 are lung cancers from second-hand smoking [10]. Survival rates for all stages and histological types are 10–15% [11].

Almost 46% of new cases of nonsmall-cell lung cancer pertain to the IIIB and IV stages [12]. In Europe, lung cancer mortality is 37.6 per 100000 people, ranging from a maximum in the UK (43.3 per 100000) to a minimum in Sweden (24.7 per 100000) [13]. In 2008, there were over 32000 new cases of lung cancer in Italy, 25147 of which were men and 6955 women, while deaths reached 26211. Not so long ago, incidence was higher in men (at a 5 : 1 ratio), but nowadays it has dropped to half (2.5/1 ratio) [14]. This malignant tumor has become more and more frequent in women due to their increasing consumption of tobacco and higher predisposition to its carcinogenic effect. In fact, trends in incidence and mortality for both sexes have been completely different with declining rates in males and increasing ones in females. Despite this hecatomb of human lives, 1.3 billion people in the world and among them 47 million Americans (25%) [15] and 11.1 million Italians over 14 years (21.7% overall; 23.9% males and 19.7% females) continue smoking [16].

## 2. The Unrestricted Rise of Lung Cancer

Tobacco smoke has been known to be harmful for health since the fifties [17, 18]. In 1889, lung cancer was an extremely rare disease: only 140 cases were registered in the world [19, 20]. Notably, a recommendation was included in the first edition of the *Merck Manual*, which was published in 1889, suggesting that smoking could be used for the treatment of bronchitis and asthma [20].

In 1912, the very first cause/effect hypothesis between lung cancer and tobacco smoking was made in a research monograph by Adler [20, 21]. In the same year, surgeon Hugh Morriston Davies carried out the first pulmonary lobectomy for lung cancer in London. The patient died of infection 8 days after the operation, due to lack of cavity draining, a procedure not followed in such cases until 1929. 

In 1914, Kellogg stated in a public health report that cancer killed 75000 people in the US each year, corresponding to 1 out of 20 deaths, and he noted that domestic animals were affected by cancer more frequently than humans, probably because of the indoor pollution deriving from combustions and tobacco smoking [22].

Almost two decades later, Dr. James Gilmore, a 48-year-old gynecologist from Pittsburgh, underwent the first successful left pneumonectomy for carcinoma. The operation was carried out by Dr. Evarts A. Graham, a pioneer in thoracic surgery [23–25]. Nearly 25 years later, Graham would die of the same disease that helped make him internationally renowned [26, 27].

Before the Second World War, experimental research on carcinogenesis from tar and polycyclic hydrocarbons was begun by an Argentinean researcher, Roffo [28–30]. Many of his studies were published in German scientific journals, which sank into oblivion after the war until WHO finally recognized him as the “forgotten father” of tobacco carcinogenesis, even though his research had already drawn the attention of tobacco manufactures in the past [31, 32].

On May 27, 1950, Ernest L. Wynder from the Sloan Kettering Institute and Evarts A. Graham published the first scientific paper on tobacco smoking as a possible etiological factor for bronchogenic carcinoma in *JAMA* [18]. 684 patients with lung cancer were studied, 96.5% of whom were heavy smokers while carcinoma was very rare (2.0%) in nonsmokers or light smokers. Wynder also assumed that 3-4 Benzopyrene, which was present in the cigarette smoke condensate, could cause cancer in humans. This hypothesis drove him to conduct the first experiments in tobacco smoke carcinogenesis.

In 1951, Richard Doll and Bradford Hill started the first extensive prospective epidemiological study, which was published in *British Medical Journal* in 1961 and confirmed the relationship between smoking and lung cancer [33]. The authors discovered that among the 1357 patients that were admitted to British hospitals with lung cancer, 99.5% were smokers.

A year later, *Reader's Digest*, which had a large circulation at the time, featured an article with the provocative title “Cancer by the Carton,” in which the role of cigarette smoke in lung cancer was described as “*a medical controversy… largely kept from public notice*.” [34] The article had an enormous impact on public opinion, putting pressure on the tobacco industry (Big Tobacco). As a consequence, on December 15, 1953, tobacco executives met at the Plaza Hotel in New York in order to create a cartel against the growing body of scientific evidence linking smoking to lung cancer, which had started to raise concern and distrust against tobacco manufacturers. Apart from the secret agreements, they jointly wrote the “*Frank Statement,*” which aimed at contrasting the evidence implicating smoking as a health issue [35]. This document/press release was published in more than 400 newspapers on January 4, 1954, reaching nearly 43 million readers. 

A decade later, the first *Surgeon General's* report that addressed the consequences of tobacco smoke for public health was released [20]. By then, the distribution of free cigarettes at annual medical and public health meetings had already stopped.

The second part of the twentieth century saw a rapid increase in this disease leading to a lung cancer epidemic, especially in males of the developed countries [36, 37]. In the US, where measures for the control of tobacco dependency had already been established in the fifties, lung cancer incidence for men peaked in 1982 and a slow but steady reduction followed afterwards [38, 39]. Conversely, in other countries, where antismoking measures were less aggressive, a similar trend has not been observed and incidence has continued rising in some countries such as Japan [40–44].

## 3. What Does the Smoker Smoke?

Even though the carcinogenetic mechanisms of tobacco smoke are not fully explored [45], only very few smokers and non-smokers know what they inhale. Tobacco smoke is a mixture of a corpuscular part (5%) and a gas phase (95%). The former, without water or nicotine, is constituted of tar. There are 0.3–3.3 billion particles per milliliter of cigarette smoke and more than 4000 compounds [46, 47], including more than 60 agents with at least sufficient evidence of carcinogenicity in laboratory animals and 11 human carcinogens according to the International Agency for Research on Cancer (IARC) [48, 49].

Besides well-known organ-specific carcinogenic substances, such as polycyclic aromatic hydrocarbons, 4-(methyl-nitrosamino)-1-(3-pyridyl)-1-butanone (NNK), 2-Naphthylaine, 4-aminobiphenyl, arsenic, and chromium, there is another one, which has recently been involved in the spy case of Litvinenko: Polonium 210 (Po-210).

## 4. Chemistry

Polonium, also called “radium F,” was discovered by Marie and Piere Curie in 1898 and was named after the home land of Curie-Sklodowska. For the discovery of radium and polonium Marie Curie received a Nobel Prize in Chemistry in 1911 [50, 51]. The element was discovered while they were investigating the cause of pitchblende's persistent radioactivity, even after the removal of uranium and radium. Their work was remarkable, considering the means available in the late nineteen century and the fact that the element can be found in uranium ores at about 0.1 mg per ton. 

Polonium is a fairly volatile metal, rarely found in nature in pitchblende containing rocks, and constitutes 2.1 × 10^−4^% of the Earth's crust [52]. The major resources of pitchblende are located in Canada, the US, Congo, and South Africa. Polonium has more than 30 radioisotopes, but Po-210 is the most dangerous and most frequent naturally occurring one [53]. This isotope has a half-life of 138.4 days, an effective biological half-time of 46 days [54], and can be created in the lab, when Bi-209 is bombarded with neutrons. It is a high energy *α*-particle emitter (5.3 MeV), but it can also emit gamma photons with energy 803 keV and emission probability of nearly 1 × 10^−5^ [55, 56]. It decays to stable Pb-206, and it has a melting point of 254°C and a boiling one of 962°C (for Pb-210 these temperatures are 327.5 and 1740°C resp.).

## 5. Toxicity

Polonium is a highly toxic element, with elevated specific radioactivity, and is dangerous to handle even in milligram amounts. The maximum allowable body burden for ingested Polonium is 1100 Bq, which is equivalent to a particle weighing only 6.6 × 10^−6^ *μ*g [57].

Alpha rays, which are formed by helium 4 (He-4) nucleus, are the least penetrating type of radiation and they manage to travel only a few centimeters in air. They can be easily stopped by obstacles, such as a sheet of paper, and they can penetrate living tissues by only a few microns [55, 58, 59]. In fact, since they lose all of their energy after a short distance, they can be dangerous for tissues only when substances emitting alpha particles enter the organism by respiration or ingestion.

In addition, alpha rays are highly ionizing and, therefore, are particularly harmful for living tissues. 1 mg of polonium can emit as many alpha particles as 5 grams of radium. The impact on humans can be devastating, as it can cause considerable damage by causing cell death, promoting a massive, progressive, and rapid necrosis, and not allowing the organism enough time to replace the quantity of dead cells [57].

## 6. Main Applications

Po-210 use is rather limited due to its high alpha radiation emissions and the difficult extraction process. The main uses are (a) as a resource of neutrons when it is mixed with beryllium, (b) as an energy resource for satellites and other space devices, (c) in antistatic devices of some precision instruments and in brushes that eliminate dust gathered on photographic film, and (d) in devices that eliminate static charges in textile mills, though less dangerous beta-ray sources are now more widely used [57].

## 7. From Earth to Tobacco

Traces of Po-210 can be found in many plants and foods and consequently, in human tissues as well [60, 61]. The principal resource of natural Po-210 is food. Spencer et al. report that 77.3% of the daily Po-210 intake of an adult male comes from food, 4.7% from water, and 0.6% from air. Notably, inhaling cigarette smoke can supply much more Po-210 (17.4%) than water and air combined [62]. 50–90% of the ingested Po-210 will promptly leave the body in feces, but the remaining fraction enters the blood circulation [63].

The discovery of the presence of Po-210 in tobacco smoke dates back to the early sixties, thanks to the work of Turner et al. [60], Marsden and Collins [64], and Radford and Hunt [65]. In fact, Po-210 and its precursor, lead 210 (Pb-210), are present in tobacco plants [66], as they may be absorbed in various associated ways.

1. Through the plant's roots, directly from terrain that contains uranium [67–69].
2. Coating on leaves as a result of meteorological events, rain, snow, and environmental dust. In fact, Radon-222, a product of U-238 decay, is a noble and volatile gas that can partially escape from terrain into the atmosphere and create Pb-210 and Po-210. These are absorbed by atmospheric dust, creating the Aitken particles that consequently lie on the leaves. The numerous trichomes of tobacco plants resemble filamentous pores and are metal accumulators, particularly of Pb-210 and Po-210. The quantity of the latter will then increase, as there is further Pb-210 decay [70, 71]. Fleischer and Parungo confirmed experimentally that radon and lead decay products are highly concentrated in the trichomes of leaves [71]. Additionally, accumulation mechanisms of Pb-210 on trichomes of tobacco have been widely discussed and studied by Martell and Poet [72, 73] while Skwarzec et al. suggested that this is the principal way Po-210 enters tobacco plants [68].
3. On the other hand, the majority of authors, such as Singh and Nilekani, have identified the importance of the fertilizers employed [74]. Calcium polyphosphates fertilizers are enriched with radium, which is chemically similar to calcium, and derive from soil that contains pitchblende and apatite [67, 75]. Interestingly, according to several studies, Indian cigarettes, which are made of scarcely fertilized tobacco, are 6 to 15 times less radioactive compared to the American ones, which derive from intensively fertilized plants [74].

## 8. From Tobacco to Lungs

The journey of Po-210 and Pb-210 towards bronchopulmonary apparatus starts by lighting a cigarette. In this combustion chamber, tobacco burns, reaching 800–900°C when inhaling, and smoke is created, which is composed of a corpuscular (5%) and a gas phase (95%) [46]. Po-210 and Pb-210 are adsorbed in the insoluble particles of the corpuscular phase [65]. The latter is present in a high quantity and is a weak alpha (<1×10^−5^), gamma, beta, and X emitter. All these inhaled particles are deposited in the broncho-pulmonary apparatus and particularly in segmental bronchi bifurcations, due to ciliary action. According to measurements by Cohen et al., radium and thorium are also present in cigarettes; however, 99% of the radioactivity comes from Po-210 [75], which remains in the broncho-pulmonary apparatus after inhalation [76].

All these particles have a different “destiny” based on the efficacy of the mucous-ciliary clearance. This mechanical purification is reduced gradually in smokers with COPD, resulting in the accumulation of insoluble Pb-210 particles, which decay to Po-210 over time [70, 77]. In fact, the more severe COPD becomes, the greater the risk of radioactive load accumulation is [77]. 

Subsequently, radioactive particles reach various organs and tissues through pulmonary and systemic circulation and cause mutations of the genetic cellular structure, deviations of the standard cellular characteristics, accelerated ageing, and quicker death due to a wide range of diseases [78, 79]. In smokers, Po-210 levels are in fact significantly higher in blood (by 30%) [65, 80], urine (6-times higher) [81], liver, kidney, heart, and psoas muscle [82]. Little and McGandy estimated that Po-210 concentration in blood is 63.64 mBq/kg of blood in smokers and 28.12 mBq/kg of blood in non-smokers [83]. Notably, concentrations of Pb-210 and Po-210 in rib bones and alveolar lung tissues were two-times higher in ex-smokers compared to non-smokers, even a year after smoking cessation [66].

Polonium radiation in the bronchial epithelium depends not only on the particle concentration of these areas, but also on the time of their permanence. Half-life of polonium is 138.38 days and of lead 22 years, which decays afterwards into polonium. There is a significant cancer risk due to chronic exposure to low levels of insoluble alpha-emitting particles [84, 85], which are responsible for high radiation doses in small tissue areas particularly in the bifurcations (hot spots) [70]. This process is facilitated by the abovementioned impaired mucous-ciliary clearance of smokers. In fact, according to Auerbach et al. [86], metaplastic lesions are present in the ciliated epithelium of all heavy smokers [87, 88]. Po-210 of the insoluble particles becomes even more penetrative because of zones with damaged or scarcely ciliated epithelium, where mucous mainly stagnates [65, 89]. More and more studies suggest that smokers and ex-smokers with moderate to severe COPD have a higher incidence of lung cancer [77, 90–92].

## 9. Po-210 Quantity in Tobacco Smoke

Po-210 alpha radioactivity in tobacco smoke depends on several variables: geographic region of tobacco growth, storage time and modality, presence of a filter, its length and composition, and the way of smoking [85]. Furthermore, the associated risk of smoke derives not only from the quantity and quality of carcinogenic substances, but also from the scarce efficacy of filters, which fail to reduce their amount adequately. In fact, common filters, found in the cigarettes of commerce, are able to reduce Po-210 activity on average by 4.6% [93]. There is evidence that resin filters may reduce lung exposure to alpha radiation even more [94].

Radford and Hunt [65] and Mussalo-Rauhamaa and Jaakkola [95] reported that about 6.5% to 22% of the Po-201 contained in cigarettes was found in mainstream smoke. Other authors stated different percentages, ranging from 3.7% to 58% [96]. According to Parfenov, approximately 50% of a cigarette's Po-210 is transferred with the smoke, 35% remains in the stub, and 15% is found in the ash [97].

Professor Gattavecchia from the Complex Unit of the Institute of Chemical, Radiochemical, and Metallurgic Sciences of University of Bologna (SMETEC), in association with ENEA (Italian National Agency for New Technologies, Energy, and Sustainable Economic Development) and the Italian Society of Tobaccology (SITAB), have conducted various studies on the alpha radioactivity of Po-210 in tobacco smoke. It has been confirmed that a cigarette with tobacco of Western origin emits 75 mBq of alpha radioactivity from Po-210, distributed in mainstream (5 mBq, 6.7%), sidestream (1.2 mBq, 1.6%), and ash (68.8 mBq, 91.7%) [97–100].

## 10. Po-210 and Second-Hand Smoking

Many studies have already reported that second-hand smoke is an important risk factor for lung cancer. After studying 91540 people for 14 years, in 1981 Hirayama demonstrated the lung cancer mortality of non-smoker wives or husbands was one-third higher compared to those with non-smoker partners [101].

This increased risk was also confirmed by a vast analysis of two case-control studies conducted in the US and Europe, in which a dose-response relationship between lung cancer risk and prolonged exposure to second-hand smoking has been found among partners, in workplaces and in public places. Risk for one-off exposure to spousal smoking increased by 18% (95% CI = 1–37%) and for long-term exposure by 23% (95% CI = 1–51%). Augmented risk for long-term exposure was also found for the work place (OR = 1.25; 95% CI = 1.03–1.51) and public places (OR = 1.26; 95% CI = 1.01–1.58) [102].

It should be considered that passive smokers are exposed to the same components as active smokers, including radioactive elements. As a matter of fact, Po-210 in second-hand smoke is 50–70% the quantity found in active smoke. Moreover, passive smokers are exposed to environmental pollution from radon, as well as from Po-210 of cigarette smoke, both of which increase lung cancer risk [87, 103, 104].

## 11. Po-210 and Narghil Smoke

Po-210 is also present in narghilé smoke. An international multidisciplinary team (from Egypt, Arabia, and France), coordinated by Khater et al., has recently published a pioneering study on narghilé (shisha, hookah) tobacco radioactivity [105]. Before this research, only very few data were available on this issue [106, 107].

The research was based on the measurement of some natural radionuclides activity and the estimation of the internal radiation dose due to narghile tobamel (moassel) smoking. Tobamel is a fashionable flavoured tobacco-molasses mixture (with added glycerol) currently used in narghilé. However, there are other forms such as jurak, similar to tobamel, but unflavoured, containing minced fruits and no glycerol [105]. It is also much stronger in nicotine. The results of the study revealed a wide range of radioactivity concentrations (in Bq/kg dry weight): U-238 = 55 Bq (19–93), Th-234 = 11 Bq (3–23), Ra-226 = 3 (1.2–8), Pb-210 = 14 Bq (3–29), Po-210 = 13 Bq (7–32), Th-232 = 7 Bq (4–10), and K-40 = 719 Bq (437–1044). The researchers concluded that the average concentrations of natural radionuclides in moassel tobacco pastes were comparable to their concentration in Greek cigarettes and tobacco leaves, and lower than that of Brazilian tobacco leaves [105].

Another recent study on the radioactivity of Greek tobacco leaves used for cigarettes showed that the annual effective dose due to inhalation by adult smokers varied from 42.5 to 178.6 *μ*Sv/y (average 79.7 *μ*Sv/y) for Ra226; 19.3 to 116.0 *μ*Sv/y (average 67.1 *μ*Sv/y) for Ra-228; 47.0 to 134.9 *μ*Sv/y (average 104.7 *μ*Sv/y) for Pb-210. In sum, the order of magnitude was the same for each radionuclide. The sum of effective doses of the three radionuclides varied from 151.9 to 401.3 *μ*Sv/y (average 251.5 *μ*Sv/y). Notably, the annual effective dose from Cs137 of Chernobyl origin was three orders of magnitude lower as it varied from 70.4 to 410.4 nSv/y (average 199.3 nSv/y) [108].

The results of Khater et al., found that the radioactivity concentration in tobacco products basically depends on the tobacco content itself, not on other ingredients such as molasses, glycerol, or fruits. Interestingly, the lower yield of Po-210 in jurak might be in relation with the Indian origin of this smoking paste. The reason might be that Po-210 alpha-radioactivity of Indian tobacco would be several times lower than that of Western tobacco [74].

## 12. Po-210 Carcinogenicity in Tobacco Smoke

Eighty-five to ninety out of a hundred lung cancers are caused by tobacco smoke; nevertheless, less than 20% of smokers get lung cancer [7]. If individuals contracting cancer on exposure to cigarette smoke are identified, the information can certainly be incorporated into effective prevention strategies [109].

Many factors could influence individual susceptibility to lung cancer in smokers. Polonium is among them, albeit it is still less considered or even ignored as a carcinogenic substance, which is also due to years of concealing by tobacco manufacturers [110]. As a matter of fact, when associated to other mutagenic and carcinogenic nonradioactive substances, which are inhaled with tobacco smoke (such as aromatic hydrocarbons, cadmium, and N-nitrosamine) [111], it seems to constitute the principal etiological factor for lung cancer [112], as long-term tissue exposure to alpha radiation can induce cancer either by itself or in association with other non-radioactive carcinogenic substances.

Polonium 210 emits alpha particles, which have a penetration limit of about 40 microns or less in animal tissue, but a very high damaging effect [55, 58, 59]. Since the late nineties, IARC has identified Po-210 as a carcinogenic element for laboratory animals, classifying it among the Group 1 agents [49].

DNA chromosome damage by exposure to alpha radiation is 100-times greater than the one caused by other types of radiation [113]. Little and Radford estimated that the radiation dose of the bronchial epithelium of bifurcations in the inferior lobes of people smoking for 25 years would be 2 Sv [114]. This can be explained by the local accumulation of Pb-210 insoluble particles [72]. According to Martell, the cumulative dose of alpha radiation in bronchial bifurcations of smokers that die of lung cancer is approximately 16 Sv (80 rad). This dose is sufficient to induce a malignant transformation caused by alpha-particles interaction with basal cells [115, 116].

Black and Bretthauer reported that Po-210 radiation dose in heavy smokers was up to 82.5 mrad (0.83 mSv) per day [117]. Radford and Hunt, estimated that the radiation dose for a person smoking two packs of cigarettes a day may be up to 0.4 Sv a year or 10 Sv over a 25-year period [65]. Such a radiation exposure dose rate was about 150-times higher than the approximately 0.05 Sv per 25 years received from natural background radiation sources. 

Many lung cancers are adenocarcinomas, a type of lung cancer that Po-210 inhalation can induce in laboratory animals [116]. Kennedy et al., induced lung cancer in hamsters, histologically similar to bronchoalveolar carcinomas (BAC) of humans, after Po-210 intratracheal instillation [118]. They also implicated the bronchiolar cell of Clara as the origin of these tumors. Moreover, according to Marmorstein, adenocarcinomas could be induced with as little as 15 rad of radioactive polonium, corresponding to one-fifth of the dose inhaled by smokers of two packets per day over a 25-year period [113].

Boffetta et al. recently reviewed seven case-control studies and estimated that the odds ratio of BAC for smoking at all was 2.47 (95% CI = 2.08–2.93). The authors also reported that the risk increased linearly with duration, amount, and cumulative cigarette smoking and persisted long after smoking cessation [119].

### 12.1. Mechanism of Action

In a recent study, Prueitt et al. tried to explain the way alpha radiations affect DNA [120]. Ionizing radiation, including Po-210, could silence the tumor suppressor gene p16(INK4a) by promoter methylation. Inactivation of this gene was found in lung cancers of both smokers and radiation-exposed non-smoker workers. The authors concluded that such inactivation was shown to play a major role in carcinogenesis, but further studies could demonstrate the level of this role compared to other carcinogenic substances.

### 12.2. Biological Harm

But what is the level of biological damage caused by tobacco smoke Po-210? Estimating the damage is a very difficult and complicated task. Using the 1990 ENEA data on the average time of Po-210 presence in lungs, which is 53 days [121], the data of the BEIR IV Committee on lung cancer risk after exposure to radon and its decay products (Pb-210, Po-210) [122], and the data of the International Commission on Radiological Protection (ICRP), which are based on the survivors of the bomb A of Hiroshima [123], it is possible to estimate the lung cancer risk, which is 4 × 10^−4^ year^−1^ (4 cases per 10000 smokers per year, which corresponds to nearly 5000 cases for the 11.1 million Italian smokers). This estimate does not take into account the promoter role of Po-210 (cocarcinogen) in the bronchopulmonary cancer and the overall carcinogenic activity of all substances [124].

To render the biological harm deriving from Po-210 in smoke more comprehensible, it has been compared to the damage caused by radiation in conventional chest X-rays. Since the dose of a modern chest radiograph is 0.034 mSv [125, 126], a smoker of 20 cigarettes per day receives a radiation dose of 0.08–0.09 Sv equivalent to approximately 300 chest X-rays per year [98, 99, 113, 127]. However, the alpha radioactivity alone does not cause the steep rise of the carcinogenic risk; instead, it is the combined and multiplicative action of each carcinogenic and co-carcinogenic component responsible for such consequence [88, 111, 128].

## 13. A Histotype Shift

There is evidence that in the last 40 years a histotype change of lung cancers has been noticed, shifting from squamous cell carcinoma to adenocarcinoma, in which the bronchial-alveolar (BAC) subtype is also included [129]. The above-mentioned shift was observed in the early seventies and has been noted ever since in the US [130, 131] and Europe [132].

The factors that have induced this shift are various and perhaps not all known. Nevertheless, almost all of them are linked to the tobacco cultivation and cigarette manufacture changes since the fifties. The most common are as follows.

- The utilization of different varieties of tobacco in the US cigarette blends. This change reduced benzopyrenes in smoke, but produced an increase of nearly 50% in nicotine-derived nitrosaminoketone (NNK) in the last quarter of the twentieth century [133, 134].
- The introduction of low-tar, low-nicotine, filtered cigarettes since the mid fifties, which seems to have contributed to the overall decline in lung cancer and the upward trend in the incidence of adenocarcinoma [135–138]. Some studies demonstrated a decline in lung cancer risk in smokers of filter cigarettes [139]. Even the common filters made of cellulose acetate have contributed to the aforementioned histotype change [140], nevertheless, smokers frequently breath in these cigarettes more deeply and as a result, a greater quantity of carcinogens is transported more distally, towards the smaller bronchial airways, where adenocarcinomas often arise [141, 142]. In addition, the increased consumption of filtered cigarettes has also reduced the yield of carcinogenic polycyclic aromatic hydrocarbons (PAHs), which are inducers of squamous cell carcinomas, simultaneously increasing the carcinogenic tobacco-specific N-nitrosamines (TSNAs), which are inducers of adenocarcinomas [137, 143].
- Interestingly, in the fifties the race for the safer filter led to a severely dangerous incident. Lorillard produced 13 billion cigarettes from 1952 through 1956 based on a filter composed of asbestos and cotton fibers. Each filter contained 10 mg of crocidolite, the fibers of which could be found in the mainstream smoke from the first two puffs. Consequently, a person smoking a 20-cigarette pack each day could inhale more than 131 million crocidolite fibers, which were longer than 5 *μ*m, in a year's time [144]. When proof of the danger of asbestos started to surface, these cigarettes were called in, after spreading harmful fibers to the lungs of thousands of smokers.
- The massive introduction of polyphosphate fertilizers in tobacco cultivations, contributing alpha radiation (from Pb-210 and Po-210) and TSNAs significantly [48], especially in Western cigarettes rather than in the ones from poor agricultural areas like India. Studies showed that American tobacco is 5.5-times more radioactive compared to the Indian-grown one (19.09 mBq/g versus 3.33 mBq/g resp.), due to the polyphosphate fertilizers [74, 145]. Because of the lower radioactivity, the prevailing type of lung cancer histotype in India is the squamous cell carcinoma and the cell type patterns have remained unchanged virtually since the early sixties [146]. As a matter of fact, in 1962, Viswanathan et al. reported 50.5% squamous cell carcinomas versus 28.4% adenocarcinomas [147] while more recent studies reported 58–67% versus 10–19%, respectively, [148–150].

## 14. How to Reduce the Radioactive Load of Tobacco Smoke?

Regulating and reducing this harmful radiation, which comes from fertilizers, could help reduce lung cancer incidence [151]. Tobacco radiation could be reduced by applying various solutions, which may also work combined.

- Use of alternative polyphosphate sources, such as organic fertilizers from animals [151].
- Use of ammonium phosphate as a fertilizer, instead of calcium phosphate [151].
- Different storage methods. A study proved that Po-210 radioactivity of tobacco rose over time while in storage [152]. As a consequence, harvesting tobacco while it is still green and avoiding prolonged storage in silos in order to prevent an increase in Po-210 concentration due to Pb-210 slow decay could be recommended.
- Genetic modifications of tobacco plants with significant reduction of trichomes concentration on the leaves, on which Pb-210 and Po-210 accumulate [71].
- Resin filters may decrease lung exposure to alpha radiation [94]. On the contrary, common filters reduce Po-210 activity, on average, by 4.6% [93].
- LaRock et al. recommended a biological way to remove Po-210 by treating polyphosphate rocks with bacteria capable of reducing sulphates [153].
- Perhaps the simplest and most applicable solutions would be the quantitative decrease in polyphosphates use in tobacco cultivations and the regulation of the maximum acceptable level of alpha radiation of cigarettes, which should also be clearly indicated on the packet [110].

## 15. Big Tobacco Has Been Aware but Kept Quiet

While multinational tobacco manufacturers have been aware of the alpha-radioactivity presence in tobacco smoke since the sixties, they have covered it up strategically. Not by chance, the polonium dossier was symbolically entitled “*waking a sleeping giant*” [110].

Among the 37 million documents that were released through the site www.pmdocs.com, one can find the lawsuit of the State of Minnesota against Philip Morris Incorporated, et al., in which there are 481 confidential documents and memorandums on the alpha-radioactivity from Po-210 in tobacco smoke (still available on 1 January 2011). The archives bring out the fact that Philip Morris has been aware of the lead and polonium existence in cigarettes since the sixties [154], as was also proved from studies by Turner et al. 1958 [60], Radford and Hunt (1964) [65], and recently by researchers from Mayo Clinic [110]. In these internal documents, it can be seen that there was a clear interest in polonium's radioactivity and the induction of bronchogenic carcinomas in laboratory animals and presumably in humans [155]. In fact, there was a recommendation to avoid any public attention to the problem for fear of “*waking a sleeping giant*” [110].

In 1980, one confidential memorandum revealed that the issue was mainly caused by calcium phosphates fertilizers employed in tobacco cultivations. Moreover, cigarette manufacturers knew about the studies conducted by Martell [72], regarding the possibility of decreasing tobacco and smoke radioactivity by using ammonium phosphate instead of calcium phosphate as fertilizer. Nevertheless, this recommendation was considered to be “an expensive point” [152].

So far, the majority of public opinion still ignores the presence of polonium radioactivity in tobacco smoke and the serious public health threat that it represents. Yet, from a communicative and motivational point of view, it could become a great opportunity for prevention and smoking cessation. For now, it seems that something has changed in the media and scientific world since the widely covered Litvinenko case and the paper of Muggli et al. [110]. In fact, the authors of the aforementioned study and the Italian Tobaccology Society have already requested the placement of a clear indication about the radioactivity content on cigarette packets.

## 16. Conclusions

Polonium-210 represents one of the principal causes of lung cancer and its shift from squamous cell carcinoma to adenocarcinoma. Provided that it is true that tobacco manufacturers have been aware of the presence of Po-210 in smoke since the early sixties and concealed its existence intentionally in various ways, it is likely that the medical and scientific sector is guilty of having ignored it.

It is necessary that the medical and scientific world becomes aware and conscious of this problem, creating systematic educational programs of tobaccology in the university curricula of the medical sciences courses. Likewise, governments should force manufacturers to introduce cigarettes with low Po-210 concentration and place a clear indication about this on the packet in order to reduce smokers' risk.

Finally, since people fear everything that is radioactive, perhaps it would be useful to create an adequate information campaign so as to enable and accelerate smokers' motivational pathways and increase the efficacy of anti-smoking programs [156].

## References

[1] Jha P, Ranson MK, Nguyen SN, Yach D (2002). Estimates of global and regional smoking prevalence in 1995, by age and sex. *American Journal of Public Health*.

[2] Makomaski Illing EM, Kaiserman MJ (2004). Mortality attributable to tobacco use in Canada and its regions, 1998. *Canadian Journal of Public Health*.

[3] World Health Organization (WHO) Cancer. http://www.who.int/cancer/en/.

[4] La Vecchia C, Bosetti C, Lucchini F (2009). Cancer mortality in Europe, 2000–2004, and an overview of trends since 1975. *Annals of Oncology*.

[5] Quinn MJ, d’Onofrio A, Moller B (2003). Cancer mortality trends in the EU and acceding countries up to 2015. *Annals of Oncology*.

[6] Blot WJ, Fraumeni JF, Schottenfeld D, Fraumeni JF (1996). Cancers of the lung and pleura. *Cancer Epidemiology and Prevention*.

[7] International Agency for Research on Cancer (IARC) (1986). Tobacco smoking. IARC monographs programme on the evaluation of the carcinogenic risk of chemicals to humans.

[8] Greenlee RT, Murray T, Bolden S, Wingo PA (2000). Cancer statistics, 2000. *Ca-A Cancer Journal for Clinicians*.

[9] World Cancer Research Fund / American Institute for Cancer Research (1997). *Food, Nutrition and the Prevention of Cancer: a Global Perspective*.

[10] Oberg M, Jaakkola MS, Woodward A, Peruga A, Pruss-Ustun A (2011). Worldwide burden of disease from exposure to second-hand smoke: a retrospective analysis of data from 192 countries. *The Lancet*.

[11] Parkin DM, Bray F, Ferlay J, Pisani P (2005). Global cancer statistics, 2002. *Ca-A Cancer Journal for Clinicians*.

[12] Mountain CF (1997). Revisions in the international system for staging lung cancer. *Chest*.

[13] European Lung Foundation Impatto in Europa. http://www.it.european-lung-foundation.org/index.php?id=446.

[14] Centro Nazionale di Epidemiologia Sorveglianza e Promozione della salute - ISS.

[15] Centers for Disease Control and Prevention (CDC) (1997). Cigarette smoking among adults—United States, 1995. *Morbidity and Mortality Weekly Report*.

[16] DOXA Il fumo in Italia. *Sintesi dei risultati*.

[17] Musk AW, de Klerk NH (2003). History of tobacco and health. *Respirology*.

[18] Wynder EL, Graham EA (1950). Tobacco smoking as a possible etiologic factor in bronchiogenic carcinoma; a study of 684 proved cases. *Journal of the American Medical Association*.

[19] Kaminsky M (1898). *Ein Primres Lungencarcinom mit Verhornten Plattenepithelien*.

[20] Borio G The tobacco timeline. 1993–2007. http://grace4life.com/History_of_Tobacco-by_Gene_Borio.pdf.

[21] Adler I (1912). *Primary Malignant Growths of the Lung and Bronchi: a Pathologic and Clinical Study*.

[22] Kellogg JH (1914). Relation of public health to race degeneraci. *American Journal of Public Health*.

[23] Graham EA (1949). The first total pneumonectomy. *Texas Cancer Bulletin*.

[24] Baue AE (1984). Landmark perspective: Evarts A. Graham and the first pneumonectomy. *Journal of the American Medical Association*.

[25] Mueller CB, Graham EA (2002). The pneumonectomy. *The Life, Lives, and Times of the Surgical Spirit of St Louis*.

[26] (1957). Death of a Surgeon. *Time*.

[27] Horn L, Johnson DH (2008). Evarts A. Graham and the first pneumonectomy for lung cancer. *Journal of Clinical Oncology*.

[28] Roffo AH (1931). Tobaccoinduced cancer in rabbits. *Zeitschrift für Krebsforschung*.

[29] Roffo AH (1936). Tobacco as a carcinogen. *Boletin del Instituto de Medicina Experimental*.

[30] Roffo AH (1939). Cancercausing benzopyrene from tobacco tar. *Zeitschrift für Krebsforschung*.

[31] Hanmer HR http://tobaccodocuments.org/atc/60359252.html.

[32] Roffo AH Carcinogenic element in various tobacco tars. http://tobaccodocuments.org/lor/85869514-9521.html.

[33] Doll R, Hill AB (1964). Mortality in relation to smoking: ten years' observations of British doctors. *The British Medical Journal*.

[34] Norr R (1952). Cancer by the carton. *Reader's Digest*.

[35] Renzi D, Renzi AH, Giorni E, Cattaruzza MS (2010). The Frank statement: the document that everyone should know. *Tabaccologia*.

[36] Bray F, Tyczynski JE, Parkin DM (2004). Going up or coming down? The changing phases of the lung cancer epidemic from 1967 to 1999 in the 15 European Union countries. *European Journal of Cancer*.

[37] Parkin DM, Bray FI, Devesa SS (2001). Cancer burden in the year 2000. The global picture. *European Journal of Cancer*.

[38] Cagle PT, Thurlbeck WM, Churg A (2005). Carcinoma of the lung. *Thurlbeck's pathology of the lung*.

[39] Jemal A, Thun MJ, Ries LA (2008). Annual report to the nation on the status of cancer, 1975–2005, featuring trends in lung cancer, tobacco use, and tobacco control. *Journal of the National Cancer Institute*.

[40] Chen F, Bina WF, Cole P (2007). Declining incidence rate of lung adenocarcinoma in the United States. *Chest*.

[41] Chen F, Cole P, Bina WF (2007). Time trend and geographic patterns of lung adenocarcinoma in the United States, 1973–2002. *Cancer Epidemiology Biomarkers and Prevention*.

[42] Sobue T, Ajiki W, Tsukuma H, Oshima A, Hanai A, Fujimoto I (1999). Trends of lung cancer incidence by histologic type: a population-based study in Osaka, Japan. *Japanese Journal of Cancer Research*.

[43] Yoshimi I, Ohshima A, Ajiki W, Tsukuma H, Sobue T (2003). A comparison of trends in the incidence rate of lung cancer by histological type in the Osaka Cancer Registry, Japan and in the surveillance, epidemiology and end results program, USA. *Japanese Journal of Clinical Oncology*.

[44] Toyoda Y, Nakayama T, Ioka A, Tsukuma H (2008). Trends in lung cancer incidence by histological type in Osaka, Japan. *Japanese Journal of Clinical Oncology*.

[45] Ames BN, Gold LS, Willett WC (1995). The causes and prevention of cancer. *Proceedings of the National Academy of Sciences of the United States of America*.

[46] Robbins Stanley L, Cotran Ramzi S (2006). *Le Basi Patologiche delle Malattie*.

[47] Stewart BW, Kleihues P (2003). International agency for research on cancer. *World Cancer Report*.

[48] Hoffmann D, Hoffmann I (1997). The changing cigarette, 1950–1995. *Journal of Toxicology and Environmental Health*.

[49] International Agency for Research on Cancer (IARC) (2004). Tobacco smoke and involuntary smoking. IARC monographs programme on the evaluation of the carcinogenic risk of chemicals to humans.

[50] Curie MP, Curie MS (1898). On a new radioactive substance contained in pitchblende. *Comptes Rendus*.

[51] http://nobelprize.org/nobel_prizes/chemistry/laureates/1911/marie-curie.html.

[52] La Sintopedia. Lainate (MI): Lombarda offset.

[53] http://en.wikipedia.org/wiki/Isotopes_of_polonium.

[54] International Agency for Research on Cancer (IARC) (2001). *Ionizing Radiation, Part 2: Some Internally Deposited Radionuclides*.

[55] Mitchell TG (1960). Some fundamentals of radiologic physics. *The Southern Medical Journal*.

[56] Kinsman S (1960). *Radiological Health Handbook*.

[57] Los Alamos National Laboratory's Chemistry Division http://periodic.lanl.gov/84.shtml.

[58] Furth J, Lorenz E, Hollaender A (1954). Carcinogenesis by ionizing radiations. *Radiation Biology*.

[59] Radford EP, Hunt VR, Little JB, Wynder EL, Hoffmann D (1969). Carcinogenicity of tobacco-smoke constituents. *Science*.

[60] Turner RC, Radley JM, Mayneord WV (1958). The naturally occurring alpha-ray activity of foods. *Health physics*.

[61] Batarekh K, Teherani DK (1987). Determination of Polonium-210 in cigarettes from Syria. *Journal of Radioanalytical and Nuclear Chemistry Letters*.

[62] Spencer H, Holtzman RB, Kramer L, Ilcewicz FH (1977). Metabolic balances of 210 Pb and 210Po at natural levels. *Radiation Research*.

[63] http://www.ead.anl.gov/pub/doc/polonium.pdf.

[64] Marsden E, Collins MA (1963). Alpha-particle activity and free radicals from tobacco. *Nature*.

[65] Radford EP, Hunt VR (1964). Polonium-210: a volatile radioelement in cigarettes. *Science*.

[66] Holtzman RB, Ilcewicz FH (1966). Lead-210 and Polonium-210 in tissues of cigarette smokers. *Science*.

[67] Tso TC, Harley NH, Alexander LT (1966). Source of Pb210 and Po210 in tobacco. *Science*.

[68] Skwarzec B, Strumińska DI, Ulatowski J, Golebiowski M (2001). Determination and distribution of 210Po in tobacco plants from Poland. *Journal of Radioanalytical and Nuclear Chemistry*.

[69] Tso TC, Hallden NA, Alexander LT (1964). Radium-226 and Polonium-210 in leaf tobacco and tobacco soil. *Science*.

[70] Rajewsky B, Stahlhofen W (1966). Polonium-210 activity in the lungs of cigarette smokers. *Nature*.

[71] Fleischer RL, Parungo FP (1974). Aerosol particles on tobacco trichomes. *Nature*.

[72] Martell EA (1974). Radioactivity of tobacco trichomes and insoluble cigarette smoke particles. *Nature*.

[73] Martell EA, Poet SE (1982). *Radon Progeny on Biological Surface and Their Effects. Bombay Simposium on Natural Radiation in the Enviroment*.

[74] Singh DR, Nilekani SR (1976). Measurement of Polonium activity in Indian tobacco. *Health Physics*.

[75] Cohen BS, Eisenbud M, Harley NH (1980). Alpha radioactivity in cigarette smoke. *Radiation Research*.

[76] Cohen BS, Eisenbud M, Wrenn ME, Harley NH (1979). Distribution of Polonium-210 in the human lung. *Radiation Research*.

[77] Schwartz AG (2006). Susceptibility to lung cancer and COPD may be genetically linked. *American Journal of Respiratory and Critical Care Medicine*.

[78] National Academy of Sciences—National Research Council (1961). *Long-Term Effects of Ionizing Radiations from External Sources*.

[79] Warren S (1956). Longevity and causes of death from irradiation in physicians. *Journal of the American Medical Association*.

[80] Shabana EI, Abd Elaziz MA, Al-Arifi MN, Al-Dhawailie AA, Al-Bokari MM (2000). Evaluation of the contribution of smoking to total blood Polonium-210 in Saudi population. *Applied Radiation and Isotopes*.

[81] Eisler H (1964). Polonium-210 and bladder cancer. *Science*.

[82] Erri ES, Baratta EJ (1966). Polonium 210 in tobacco, cigarette smoke, and selected human organs. *Public Health Reports*.

[83] Little JB, McGandy RB (1966). Measurement of Polonium-210 in human blood. *Nature*.

[84] Karali T, Ole S, Veneer G (1996). Study of spontaneous deposition of 210Po on various metals and application to activity assessment in cigarette smoke. *Applied Radiation and Isotopes*.

[85] Watson AP (1985). Polonium-210 and lead-210 in food and tobacco products: transfer parameters and normal exposure and dose. *Nuclear Safety*.

[86] Auerbach O, Stout AP, Hammond EC, Garfinkel L (1962). Bronchial epithelium in former smokers. *The New England Journal of Medicine*.

[87] Winters TH, DiFranza JR (1982). Radioactivity in cigarette smoke (letter). *The New England Journal of Medicine*.

[88] Little JB, McGandy RB, Kennedy AR (1978). Interactions between Polonium-210 alpha-radiation, benzo(a)pyrene, and 0.9% NaCl solution instillations in the induction of experimental lung cancer. *Cancer Research*.

[89] Cohen BS, Harley NH, Tso TC (1985). Clearance of Polonium-210-enriched cigarette smoke from the rat trachea and lung. *Toxicology and Applied Pharmacology*.

[90] Bach PB, Kattan MW, Thornquist MD (2003). Variations in lung cancer risk among smokers. *Journal of the National Cancer Institute*.

[91] Skillrud DM, Offord KP, Miller RD (1986). Higher risk of lung cancer in chronic obstructive pulmonary disease. A prospective, matched, controlled study. *Annals of Internal Medicine*.

[92] Van den Eeden SK, Friedman GD (1992). Forced expiratory volume (1 second) and lung cancer incidence and mortality. *Epidemiology*.

[93] Skwarzec B, Ulatowski J, Struminska DI, Borylo A (2001). Inhalation of 210Po and 210Pb from cigarette smoking in Poland. *Journal of Environmental Radioactivity*.

[94] Bretthauer EW, Black SC (1967). Polonium-210: removal from smoke by resin filters. *Science*.

[95] Mussalo-Rauhamaa H, Jaakkola T (1985). Plutonium-239, 240Pu and 210Po contents of tobacco and cigarette smoke. *Health Physics*.

[96] Khater AE (2004). Polonium-210 budget in cigarettes. *Journal of Environmental Radioactivity*.

[97] Parfenov YD (1974). Polonium-210 in the environment and in the human organism. *Atomic Energy Review*.

[98] Zagà V, Gattavecchia E (2006). Polonio 210 nel fumo di tabacco: il killer radioattivo. *Tabaccologia*.

[99] Gattavecchia E, Chiamulera C, Zagà V Alpha radioactivity, Polonium 210 and tobacco smoke.

[100] Zagà V, Gattavecchia E (2008). Poloniul: ucigasul radioactiv din fumul de tutun. *Pneumologia*.

[101] Hirayama T (1981). Non-smoking wives of heavy smokers have a higher risk of lung cancer: a study from Japan. *British Medical Journal (Clinical Research Ed)*.

[102] Brennan P, Buffler PA, Reynolds P (2004). Secondhand smoke exposure in adulthood and risk of lung cancer among never smokers: a pooled analysis of two large studies. *International Journal of Cancer*.

[103] Winters TH, DiFrenza J (1983). Radioactivity and lung cancer in active and passive smokers. *Chest*.

[104] Stone R (1992). Bad news on second-hand smoke. *Science*.

[105] Khater AE, Abd El-Aziz NS, Al-Sewaidan HA, Chaouachi K (2008). Radiological hazards of Narghile (hookah, shisha, goza) smoking: activity concentrations and dose assessment. *Journal of Environmental Radioactivity*.

[106] Abdul-Majid S, Kutbi II, Basabrain M (1995). Radioactivity levels in jurak and moasel, comparison with cigarette tobacco. *Journal of Radioanalytical and Nuclear Chemistry*.

[107] Al-Arifi MN (2005). Estimating of the amount of 210Po released with the smoke stream into smoker's lung from cigarette tobacco and some smoking pastes in Saudi Arabia. *Journal of Medical Sciences*.

[108] Papastefanou C (2009). Radioactivity of tobacco leaves and radiation dose induced from smoking. *International Journal of Environmental Research and Public Health*.

[109] Hecht SS (2002). Cigarette smoking and lung cancer: chemical mechanisms and approaches to prevention. *Lancet Oncology*.

[110] Muggli ME, Ebbert JO, Robertson C, Hurt RD (2008). Waking a sleeping giant: the tobacco industry’s response to the Polonium-210 issue. *American Journal of Public Health*.

[111] Kilthau GF (1996). Cancer risk in relation to radioactivity in tobacco. *Radiologic Technology*.

[112] Martell EA (1983). Alpha-radiation dose at bronchial bifurcations of smokers from indoor exposure to radon progeny. *Proceedings of the National Academy of Sciences of USA*.

[113] Marmorstein J (1986). Lung cancer: is the increasing incidence due to radioactive Polonium in cigarettes?. *Southern Medical Journal*.

[114] Little JB, Radford EP (1967). Polonium-210 in bronchial epithelium of cigarette smokers. *Science*.

[115] Martell EA (1982). Radioactivity in cigarette smoke (correspondence). *The New England Journal of Medicine*.

[116] Martell EA, Sweder KS The roles of Polonium isotopes in the etiology of lung cancer in cigarette smokers and uranium miners.

[117] Black SC, Bretthauer EW (1968). Polonium-210 in tobacco. *Radiological health data and reports*.

[118] Kennedy AR, McGandy RB, Little JB (1977). Histochemical, light and electron microscopic study of Polonium-210 induced peripheral tumors in hamster lungs: evidence implicating the Clara cell as the cell of origin. *European Journal of Cancer and Clinical Oncology*.

[119] Boffetta P, Jayaprakash V, Yang P (2011). Tobacco smoking as a risk factor of bronchioloalveolar carcinoma of the lung: pooled analysis of seven case-control studies in the International Lung Cancer Consortium (ILCCO). *Cancer Causes Control*.

[120] Prueitt RL, Goodman JE, Valberg PA (2009). Radionuclides in cigarettes may lead to carcinogenesis via p16 inactivation. *Journal of Environmental Radioactivity*.

[121] (1990). *Livelli di Riferimento per La Contaminazione Interna*.

[122] National Research Council (U.S.). Committee on the Biological Effects of Ionizing Radiations., United States. Environmental Protection Agency., U.S. Nuclear Regulatory Commission (1988). *Health Risks of Radon and Other Internally Deposited Alpha-Emitters*.

[123] (1991). 1990 Recommendations of the International Commission on Radiological Protection. *Annals of the ICRP*.

[124] Hoffmann D, Hoffmann I, El-Bayoumy K (2001). The less harmful cigarette: a controversial issue. A tribute to Ernst L. Wynder. *Chemical Research in Toxicology*.

[125] Pillay M, Sampson CB (1990). Dosimetric aspect. *Textbook of Radiopharmacy*.

[126] Johns HE, Cunningham JR (1983). *The Physics of Radiology*.

[127] Winters TH, Di Franza JR (1982). Radioactivity in cigarette smoking. *The New England Journal of Medicine*.

[128] Little JB, O'Toole WF (1974). Respiratory tract tumors in hamsters induced by benzo(a)pyrene and 210Po alpha-radiation. *Cancer Research*.

[129] Vincent RG, Pickren JW, Lane WW (1977). The changing histopathology of lung cancer: a review of 1682 cases. *Cancer*.

[130] Anton-Culver H, Culver BD, Kurosaki T, Osann KE, Lee JB (1988). Incidence of lung cancer by histological type from a population-based registry. *Cancer Research*.

[131] Wingo PA, Ries LA, Giovino GA (1999). Annual report to the nation on the status of cancer, 1973–1996, with a special section on lung cancer and tobacco smoking. *Journal of the National Cancer Institute*.

[132] Devesa SS, Bray F, Vizcaino AP, Parkin DM (2005). International lung cancer trends by histologic type: male:female differences diminishing and adenocarcinoma rates rising. *International Journal of Cancer*.

[133] Stellman SD, Muscat JE, Hoffmann D, Wynder EL (1997). Impact of filter cigarette smoking on lung cancer histology. *Preventive Medicine*.

[134] Boyle P (2004). *Tobacco and Public Health : Science and Policy*.

[135] Peto R (1986). Overview of cancer time-trend studies in relation to changes in cigarette manufacture. *IARC Scientific Publications*.

[136] Stellman SD, Muscat JE, Thompson S, Hoffmann D, Wynder EL (1997). Risk of squamous cell carcinoma and adenocarcinoma of the lung in relation to lifetime filter cigarette smoking. *Cancer*.

[137] Wynder EL, Hoffmann D (1994). Smoking and lung cancer: scientific challenges and opportunities. *Cancer Research*.

[138] Wynder EL, Muscat JE (1995). The changing epidemiology of smoking and lung cancer histology. *Environmental Health Perspectives*.

[139] Benhamou S (1993). Cancers related to tobacco smoking. *Revue du Praticien*.

[140] Ito H, Matsuo K, Tanaka H (2010). Nonfilter and filter cigarette consumption and the incidence of lung cancer by histological type in Japan and the United States: analysis of 30-year data from population-based cancer registries. *International Journal of Cancer*.

[141] Wynder EL, Hoffmann D (1998). Re: cigarette smoking and the histopathology of lung cancer. *Journal of the National Cancer Institute*.

[142] Djordjevic MV, Hoffmann D, Hoffmann I (1997). Nicotine regulates smoking patterns. *Preventive Medicine*.

[143] International Agency for Research on Cancer (IARC) (2004). Tobacco smoke and involuntary smoking. IARC monographs programme on the evaluation of the carcinogenic risk of chemicals to humans.

[144] Longo WE, Rigler MW, Slade J (1995). Crocidolite asbestos fibers in smoke from original kent cigarettes. *Cancer Research*.

[145] Harley NH, Cohen BS, Tso TC, Moghissi AA, Paras P, Carter MW, Barker RF (1978). Polonium-210 in tobacco. *Radioactivity in Consumer Products*.

[146] Behera D, Balamugesh T (2004). Lung cancer in India. *The Indian Journal of Chest Diseases & Allied Sciences*.

[147] Viswanathan R, Sen G, Iyer PV (1962). Incidence of primary lung cancer in India. *Thorax*.

[148] Thippanna G, Venu K, Gopalakrishnaiah V, Reddy PN, Sai Charan BG (1999). A profile of lung cancer patients in hyderabad. *Journal of the Indian Medical Association*.

[149] Gupta D, Boffetta P, Gaborieau V, Jindal SK (2001). Risk factors of lung cancer in Chandigarh, India. *Indian Journal of Medical Research*.

[150] Kashyap S, Mohapatra P, Negi R (2003). Pattern of primary lung cancer among bidi smokers in North-Western Himalayan region of India. *Lung Cancer*.

[151] ACSA Radioactive Polonium found in tobacco. http://www.acsa2000.net/HealthAlert/lungcancer.html.

[153] LaRock P, Hyun JH, Boutelle S, Burnett WC, Hull CD (1996). Bacterial mobilization of Polonium. *Geochimica et Cosmochimica Acta*.

[154] Seligman RB Polonium 210 (Reference R.D. Carpenter to DR. H. Wakeman and DR. R.B. Seligman, 651216, Polonium in tobacco).

[155] Carpenter RD Polonium in tobacco. Interoffice memorandum.

[156] Simpson D (2007). UK: pulling the Polonium ad. *Tobacco Control*.

